# Environmental Dynamics as a Structuring Factor for Microbial Carbon Utilization in a Subtropical Coastal Lagoon

**DOI:** 10.3389/fmicb.2013.00014

**Published:** 2013-02-15

**Authors:** Cecilia Alonso, Claudia Piccini, Fernando Unrein, Florencia Bertoglio, Daniel Conde, Jakob Pernthaler

**Affiliations:** ^1^Functional Ecology of Aquatic Systems, Centro Universitario Región Este, Universidad de la RepúblicaRocha, Uruguay; ^2^Instituto de Investigaciones Biológicas Clemente EstableMontevideo, Uruguay; ^3^Instituto de Investigaciones Biotecnológicas-Instituto Tecnológico de Chascomús, Consejo Nacional de Investigaciones Científicas y Técnicas-Universidad Nacional de San MartínBuenos Aires, Argentina; ^4^Facultad de Ciencias, Universidad de la RepúblicaMontevideo, Uruguay; ^5^Limnological Station, Institute of Plant Biology, Zurich UniversityKilchberg, Switzerland

**Keywords:** bacterioplankton, carbon, estuary, Laguna de Rocha, hydrology

## Abstract

Laguna de Rocha belongs to a series of shallow coastal lagoons located along South America. It is periodically connected to the sea through a sand bar, exhibiting a hydrological cycle where physicochemical and biological gradients are rapidly established and destroyed. Its most frequent state is the separation of a Northern zone with low salinity, high turbidity and nutrient load, and extensive macrophyte growth, and a Southern zone with higher salinity and light penetration, and low nutrient content and macrophyte biomass. This zonation is reflected in microbial assemblages with contrasting abundance, activity, and community composition. The physicochemical conditions exerted a strong influence on community composition, and transplanted assemblages rapidly transformed to resembling the community of the recipient environment. Moreover, the major bacterial groups responded differently to their passage between the zones, being either stimulated or inhibited by the environmental changes, and exhibiting contrasting sensitivities to gradients. Addition of allochthonous carbon sources induced pronounced shifts in the bacterial communities, which in turn affected the microbial trophic web by stimulating heterotrophic flagellates and virus production. By contrast, addition of organic and inorganic nutrient sources (P or N) did not have significant effects. Altogether, our results suggest that (i) the planktonic microbial assemblage of this lagoon is predominantly carbon-limited, (ii) different bacterial groups cope differently with this constraint, and (iii) the hydrological cycle of the lagoon plays a key role for the alleviation or aggravation of bacterial carbon limitation. Based on these findings we propose a model of how hydrology affects the composition of bacterioplankton and of carbon processing in Laguna de Rocha. This might serve as a starting hypothesis for further studies about the microbial ecology of this lagoon, and of comparable transitional systems.

## Introduction

Coastal lagoons are continental shallow and brackish water bodies partially or completely separated from the ocean by barriers of sand or coral (Woodroffe, 2002). From a hydrodynamic viewpoint their hydrology mixes characteristic features of shallow lakes, reservoirs, and rivers, which greatly determines ecosystem functioning (Mann, 2000). When coastal lagoons are connected to the ocean strong physicochemical gradients are generated especially in terms of salinity, light, and nutrient availability (Hauenstein and Ramírez, 1986). These transitional aquatic systems are extremely complex and characterized by high primary productivity, which in turn supports high secondary production (Viaroli et al., 1996). They are also sites of high biological diversity at all levels, from micro-organisms (Thompson et al., 2011) to birds (Aldabe et al., 2006), interacting through a complex food web with high adaptation potential and resilience capacity (Milessi et al., 2010). Consequently, coastal lagoons provide numerous ecosystem services such as water quality maintenance, fish production, carbon fixation and transformation, and protection from erosion (Gönenç and Wolflin, 2005).

In general, transitional coastal systems are particularly relevant for the global carbon cycle due to their disproportionally high productivity and participation in C turnover (compared to their size), and their linking of the terrestrial and marine phases of biogeochemical cycles (Schlesinger, 1997). Coastal lagoons, being shallow and strongly exposed to human activity, represent one of the ecosystem types most sensitive to external perturbations. Examples of current challenges for these systems include anthropogenic eutrophication, the alteration of the evaporation/precipitation balance due to climate change, wetland area loss, the local extinction of native species, and the introduction of exotic species (Meerhoff et al., 2012). Thus, studies on the ecological functioning of coastal lagoons are essential to better understand the responses of these vulnerable aquatic systems to environmental change (Brito et al., 2012; Meerhoff et al., 2012).

Laguna de Rocha belongs to a series of coastal shallow lagoons located along the south-eastern line of South America. This large water body is periodically connected to the sea through a sand bar, exhibiting a hydrological cycle with three phases of rapid establishment and destruction of physicochemical and biological gradients (Conde et al., 2000). During the first phase, characterized by dominance of freshwater discharge, the lagoon exhibits homogeneous physicochemical characteristics: high loads of dissolved and particulate organic and inorganic components, high values of chl*a* and primary production, and brackish oligohaline conductivity values between 3 and 8 mS cm^−1^. This phase usually lasts between 3 weeks and 3 months, until the increased water level leads to an overflow of the sand bar, and consequently to discharge into the Atlantic Ocean. In phase II, the ocean intrudes into the lagoon, usually under the influence of south-western winds. With the progression of the salinity gradient the Southern area of the lagoon shifts to a brackish meso- to polyhaline condition (10–45 mS cm^−1^), characterized by low nutrient content, high UV penetration, low chl*a* and primary production, while the Northern zone remains brackish oligohaline (3–8 mS cm^−1^). This phase is the most frequent state of the lagoon and typically lasts between 1 and 2 weeks and 4 months. Incessant south-western winds can then move saline waters further North (Phase III), so that most of the lagoon turns to brackish meso- to polyhaline condition and the associated characteristics. This phase is not very frequently attained, and typically lasts for a comparably short period (1–3 weeks; Conde et al., 2000).

The hydrological cycle in Laguna de Rocha exerts a strong effect on different planktonic communities (Bonilla et al., 2005; Calliari et al., 2009; Britos, 2010), including the bacterioplankton. The pelagic microbial assemblages of the lagoon exhibit high abundances, productivity and diversity (Piccini et al., 2006; Thompson et al., 2011), and all these parameters are affected by the changes in environmental conditions during the different phases of the hydrological cycle (Piccini et al., 2006). However, so far there is not sufficient information to postulate a model of how hydrology affects the composition and functioning of bacterioplankton in this lagoon, in particular focusing on the microbial carbon cycling.

The aim of this paper is to synoptically present and discuss results from three experiments about the interactions between bacterial communities, substrate and nutrient availability, and the hydrological dynamics of the lagoon. Specifically, we assessed the responses of bacterial assemblages to environmental change, and we examined aspects of bacterial carbon utilization and limitation patterns (growth, biomass production, and transfer to other trophic levels). Based on these observations, we propose a model for the functioning of the relationship between bacterioplankton and hydrology, which may serve as a framework for further studies both in the lagoon and in comparable transitional systems.

## Materials and Methods

### Study site

Laguna de Rocha is a shallow, highly productive, and large brackish lagoon (mean depth, 0.6 m; area, 72 km^2^), located in Uruguay, on the south-eastern coast of South America (34°33’S, 54°22’W) and part of a MaB/UNESCO Biosphere Reserve. This lagoon is influenced by freshwater from the drainage of three streams, Rocha stream (RS) being the most important one in terms of flow, and by periodic marine intrusions through a single mouth inlet from the Atlantic Ocean across a sand barrier.

This intercommunication of the lagoon with the ocean may occur several times per year and the water discharge of the lagoon during such events may reach up to 570 m^3^ s^–1^ (Conde and Sommaruga, 1999). Aerial views of the closed and open situations can be found at http://zoology.uibk.ac.at/limno/images/rocha.html. Relevant limnological information about the system has been published elsewhere (Conde and Sommaruga, 1999; Conde et al., 1999).

### Experiments

Three independent sets of experiments were conducted to investigate the bacterial response to the hydrologic dynamics of the lagoon: A full transplant of bacterial communities between sites (Experiment 1), a gradual mixing of bacterial communities mimicking an environmental gradient (Experiment 2), and a manipulation of carbon and nutrient sources in a mesocosms (Experiment 3).

#### Experiment 1. Transplant of bacterial communities across environmental gradients

Sites for sampling and incubation of bacterial assemblages were selected based on their physicochemical characteristics at the time of the experiment (freshwater: Rocha stream [RS]; brackish water: Northern region of Laguna de Rocha [LN]). Conductivity was measured *in situ* with a portable meter (ES-12; Horiba Inc., Irvine, CA, USA). Water samples were analyzed for ammonium, nitrite, nitrate, and total nitrogen, soluble reactive phosphorus, total phosphorus, suspended solids, and organic matter content according to standard methods (APHA, 1995).

##### Experimental set up

Water samples from both chosen sites were collected in acid-washed 20 l plastic carboys in November 2004 (Southern springtime). Half of each sample was filtered through 0.8 μm pore-size filters to produce grazer-free (−G) treatments, whereas the original grazer community was maintained in the unfiltered second half of the samples (+G). The filtered and unfiltered water samples from both sites were placed in triplicate sets of pre-treated (acid-washed and deionized water rinsed) dialysis bags (diameter, 75 mm; molecular weight cut-off, 12,000–16,000 Da; Poly Labo, Switzerland), that were cut into pieces of 50 cm length to hold 2 l of water. A first set was filled with water (either for +G or −G treatments) from RS and incubated at RS (“freshwater incubation,” RS–RS), another set was filled with water from LN and incubated at LN (“brackish water incubation,” LN–LN). A third set bags was filled with water from RS and incubated at LN (“transplanted incubation,” RS-LN) and a fourth set was filled with a 50:50 mix of water from RS and LN and incubated at LN (“mix incubation,” Mix-LN). Samples were taken at 24, 48, and 72 h of incubation, and bacterial abundance, activity, and community composition were determined (see below). Conductivity (*K*) inside the dialysis bag was used as a proxy of changes in the main environmental characteristics (Conde et al., 1999).

##### Bacterial abundances

Portions of 3 ml were taken from each dialysis bag at each sampling, fixed with 2% (v/v) paraformaldehyde (PFA), incubated at room temperature in the dark for 1 h and then stored at −20°C until further analysis. The PFA-fixed samples were stained with 4,6′-diamino-2-phenylindole (DAPI; final concentration, 1 μg ml^−1^), filtered onto polycarbonate filters (diameter 25 mm; pore-size, 0.2 μm; type GTTP; Millipore, and 1000 bacterial cells per sample were counted on an epifluorescence microscope (Axioplan II Imaging; Carl Zeiss, Jena, Germany).

##### Bacterial production

Bulk bacterial activity was estimated from the incorporation of [^3^H]-l-leucine (Amersham, Little Chalfont, England) into bacterial biomass (Simon and Azam, 1989). Sample pools were made with triplicates, using *in situ* samples and samples taken from the dialysis bags after 72 h. Radiolabeled leucine (specific activity 63 Ci mmol^−1^) was added at saturating concentrations (20 nM) to three sub-samples of each pool and to one formaldehyde-fixed control (final concentration, 3% v/v) from each water treatment (Piccini et al., 2006). These sub-samples were incubated in a water bath in the dark at *in situ* temperature (18.5°C) for 1 h and then fixed by the addition of formaldehyde. Macromolecule extraction was done with ice-cold TCA (5% w/v) and ethanol (80% v/v) as described previously (Simon and Azam, 1989). [^3^H]-l-leucine incorporation was determined with a Beckman LS5000TD liquid scintillation counter (Beckman, Fullerton, CA, USA). Values were corrected for quenching (external standard method) and by subtraction of counts from the controls. Leucine incorporation was converted to bacterial protein and carbon production using published conversion factors (Simon and Azam, 1989).

##### Bacterial community composition

PFA-fixed bacteria from 3 ml of sample were filtered onto white 0.2 μm pore-size polycarbonate filters (diameter 47 mm). The filters were rinsed twice with 1× phosphate buffered saline (PBS) and once with distilled water and stored at −20°C. FISH with horseradish-peroxidase labeled probes and tyramide signal amplification (CARD-FISH) was performed as described previously (Piccini et al., 2006), see Table A1 in Appendix for details on the targeted groups. After hybridization the samples were counterstained with DAPI (1 μg ml^−1^). The relative abundance of each targeted group was determined by epifluorescence microscopy and semi-automated counting (Pernthaler et al., 2003).

#### Experiment 2. Mixing of bacterial communities (simulation of environmental gradient)

Water samples were obtained from two sites (LN and LS) selected according to their physicochemical characteristics at the time of the experiment (July 2007; Southern winter). Conductivity was measured *in situ* with a portable meter (ES-12; Horiba Inc., Irvine, CA, USA). Water samples were analyzed for ammonium, nitrite, nitrate, soluble reactive phosphorus, according to standard methods (APHA, 1995).

##### Experimental set up

Water samples from both sites were mixed in different proportions (% of water from LN to % of water from LS: 100:0, 90:10, 75:25, 50:50, 25:75, 10:90, 0:100) to a final volume of 500 ml. Each treatment was performed in triplicates. Immediately after mixing, triplicate 10 ml sub-samples plus one PFA pre-fixed control were taken from each variant. Subsequently, l-[4,5-^3^H]Leucine (Amersham, specific activity 2.26 TBq mmol^−1^) was added to these sub-samples at a final concentration of 10 nM. The incubations were run for 8 h in the dark at ambient water temperature (6°C) and stopped by addition of PFA (final concentration, 1%). Half of each sample was used to estimate bulk bacterial carbon production (BCP, applying the same protocol as for experiment 1), whereas the remaining volume served to trace the incorporation of the tracer by individual bacterial cells (see below).

##### Tracer incorporation by specific bacterial groups

After fixation all samples were filtered through polycarbonate filters (type GTTP, pore-size, 0.2 μm, diameter 25 mm, Millipore, Eschborn, Germany). The filters were rinsed twice with sterile phosphate buffered saline (PBS) and stored at −20°C until further analysis. To study the substrate uptake by specific bacterial groups we combined microautoradiography (MAR) and CARD-FISH as described previously (Alonso and Pernthaler, 2005). Triplicate samples of every treatment type were evaluated per bacterial group. Different MAR exposure times (4 h to 3 days) were tested to produce a maximum number of cells with silver grains (Alonso, 2012), and an optimal exposure time of 18 h was determined. All photochemicals were purchased from Kodak (Eastman Kodak, Rochester, NY, USA): autoradiography emulsion (type NTB-2), developer (type Dektol), and fixer. The development of the exposed slides followed the instructions of the manufacturer.

##### Bacterial community composition

The abundances of different bacterial populations were determined by CARD-FISH as described above (see Table A1 in Appendix for details on the targeted groups). CARD-FISH and MAR-FISH preparations were embedded on microscopic slides in a previously described mounting medium containing 4,6′-diamidino-2-phenylindole (DAPI, final concentration, 1 μg ml^−1^). Evaluation was carried out following the strategy outlined in Pernthaler et al. (2003) and Alonso and Pernthaler (2005) on a motorized microscopic system consisting of an epifluorescence microscope (AxioImager.Z1, Zeiss, Germany), a CCD Camera (Zeiss AxioCam MRm), and a motorized stage for eight microscopic slides (Zeiss WSB Piezodrive 05). Automation was achieved using the Visual Basic for Application module of the AxioVision software (Carl Zeiss) and comprised automated sample recognition and localization, multi-channel image acquisition, image processing, and cell counting routines for both FISH and MAR-FISH preparations (Zeder, 2010). A manual verification of the results of automated counting in a subset of preparations was assisted by the free counting software “ClickCounter” (http://www.technobiology.ch).

#### Experiment 3. Manipulation of carbon and nutrient sources in mesocosms

Two sampling sites were selected according to their physicochemical characteristics at the time of the experiment (December 2008, Southern late spring). Freshwater was obtained from Rocha stream (RS), whereas the brackish water site in the Northern part of Laguna de Rocha (LN) served as the main location for sampling and incubations. Conductivity was measured *in situ* with a portable meter (ES-12; Horiba Inc., Irvine, CA, USA).

##### Experimental set up

Water samples from LN were distributed among six sets of triplicate mesocosms (PVC tubes, 4.5 l) that represented different treatments with respect to added carbon and nutrient sources. Nitrogen was added in the form of either NH_4_Cl (Treatment 1), or urea (Treatment 2) at a final *N* concentration of 150 μg l^−1^. Phosphorous was added to Treatment 3 as KH_2_PO_4_ at a final *P* concentration of 50 μg l^−1^. To treatment 4 a macrophyte concentrate was added, which was prepared as follows: Five submerged stems of *S. californicus* (15–20 cm length) from the lagoon were homogenized using a lab blender and 500 ml of sterile Milli-Q water. Homogenates were pre-filtered through a 50 μm mesh to remove debris. The resulting crude extract was sterilized by filtration through 0.2 μm polycarbonate filters (Millipore) that had previously been soaked overnight in 10% HCl and rinsed with Milli-Q water. The plant extract was then added to a final concentration of 10% (v/v). Treatment 5 consisted of a 50:50 mix of LN and RS water, and Treatment 6 was a control of unmodified LN water.

The incubations were run for 42 h under *in situ* light and temperature conditions. Samples were taken at the beginning (t_0_) and at regular intervals during the incubations (4, 8, 16, 24, 32, and 42 h) in order to follow (i) the biomass and composition of primary producers, (ii) bacterial abundance and the relative proportions of bacteria with different nucleic acid content, (iii) abundance of main bacterial groups, (iv) the abundances and predatory activity of bacterial grazers, and (v) viral abundances (see Table A2 in Appendix for details on which samples were analyzed by which methods).

##### Biomass of primary producers

The relative amounts of phytoplankton chlorophyll-a (Chl*a*) and phycocyanin (PC) were estimated *in vivo* using an AquaFluor (Turner design) fluorometer.

##### Abundances of bacteria and picocyanobacteria

Bacterial and picocyanobacterial abundances were determined by flow cytometry. Samples for heterotrophic bacteria were stained with a dilution of dimethyl sulfoxide-Syto13 (Molecular Probes) at 2.5 mM, while picocyanobacterial abundance was determined from unstained portions of the same samples. All evaluations were done with a CyAn™ ADP analyzer equipped with a 488 nm laser. Bacteria and picocyanobacteria were detected in cytometric biplots of 90° light scatter (SSC) vs. green (FL1) and red (FL3) fluorescence, respectively (Gasol et al., 1999). Three different populations of heterotrophic bacteria with contrasting content of nucleic acids (HNA, MNA, and LNA) were distinguished according to the intensity of the green fluorescence signal.

##### Abundance of main bacterial groups

Based on the data on bacterial abundance and proportions of HNA and LNA cells determined by flow cytometry, a subset of samples was selected for analyzing bacterial community composition by CARD-FISH and automated microscopy as described above (see Table A1 in Appendix for details on the targeted groups).

##### Abundance and bacterivory of protistan grazers

Estimates of grazing rates by heterotrophic flagellates (HF) on bacteria were determined from the ingestion of fluorescently labeled bacteria (FLB; Sherr and Sherr, 1993). FLB were prepared as previously described (Sherr and Sherr, 1987) from a *Brevundimonas diminuta* (syn. *Pseudomonas diminuta*) strain obtained from the Spanish Type Culture Collection (Burjassot, València) and kept frozen (−20°C) until use. The size of FLB was assessed by epifluorescence microscopy and using image analysis (mean cell volume ± 1 SD: 0.083 ± 0.053 μm^3^).

Grazing experiments were performed in sub-samples of 30 ml from each treatment at t0, t3, and t6. Tracers were added at about 20% of natural bacterial concentrations. Samples were fixed after 15 min of incubation with 4% cold glutaraldehyde (2%, final concentration), filtered through a 0.8 μm pore-size polycarbonate filter (Nucleopore, Whatman) and stored at −20°C until analyzed. Prior to the processing of filters by epifluorescence microscopy they were mounted on slides with a drop of Vectashield immersion fluid (Vecta Laboratories Inc.) mixed with DAPI. HF were enumerated at 10,040× magnification, and ingested FLB were counted at the same time. A mean of 80 HF were inspected on each filter, yielding about 30 ingested FLB. Ingestion rates (FLB HF^−1^ h^−1^), clearance rates (nl HF^−1^ h^−1^), and specific grazing rates (SGR, bacteria HF^−1^ h^−1^) were estimated as described before (Unrein et al., 2007). Grazing impact on bacteria was estimated by multiplying SGR by the HF abundance (bacteria ml^−1^ h^−1^). Bacterial turnover rates (% day^−1^) were estimated by expressing the extrapolated daily grazing impact as a percentage of the corresponding bacterial abundance. Bacterial net growth rate (h^−1^) was calculated by dividing the change in bacterial abundance (Ln transformed) between t6 and t0 by the incubation time. Net bacterial production (NBP; bact. ml^−1^ h^−1^) was estimated by multiplying the net growth rate by the bacterial abundance at t0.

##### Viral abundance

Samples for virus counting were fixed with PFA at the final conc. of 1% and stored at −80°C until analyzed. Fixed samples were filtered onto 0.02 μm pore-size Anodisc filter and stained with SYBR Gold according to Chen et al. (2001). Viral counting was done using an inverted epifluorescence microscope (Olympus IX81), at least 1000 SYBR Gold-stained viral-sized particles were counted for each sample (Chen et al., 2001; Patel et al., 2007).

##### Statistical analyses

All statistical analyses were performed using GraphPad Prism version 5.0 for Mac OS X, GraphPad Software (San Diego, CA, USA). If required, data were log- or arcsine- transformed prior to analyses in order to approximate normality (Kolmogorov–Smirnov test); if this was not obtainable, parametric tests were replaced by their non-parametric alternatives such as Mann–Whitney U test for paired comparisons (M–W) and Kruskal–Wallis ANOVAs for multiple comparisons (K–W). *Post hoc* comparisons between samples after ANOVA were performed using Bonferroni tests. For experiment 1, a one-way ANOVA was performed to determine if differences in BCP between treatments were significant (*P* ≤ 0.05). Differences in bacterial abundances between incubation times were analyzed by K–W followed by M–W for paired comparison. The differences between the relative abundance of each bacterial group between incubation times and treatments were analyzed using two-way ANOVAs. For experiment 2, differences in bacterial abundance and carbon production between treatments were analyzed by one-way ANOVAs. The differences between treatments in the relative abundances of bacterial groups and of active bacteria were analyzed by two-way ANOVAs. For experiment 3, the differences in chl*a* concentration, cyanobacterial, and bacterial abundance among treatments and at different incubation times were analyzed by two-way ANOVAs.

## Results

### Experiment 1. Full transplant of bacterial communities

#### Chemical characteristics of water at the study sites and in the dialysis bags

**Figure A1:**
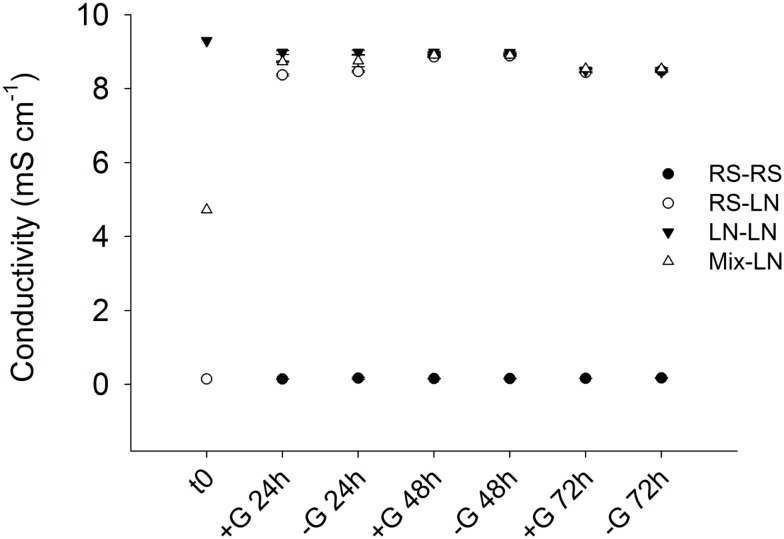
**Exp. 1, Evolution of conductivity inside the dialysis bags along incubation time**.

In parallel with higher conductivity, almost six times lower NO_3_ concentrations were observed in LN than in RS (Table A3 in Appendix), whereas total nitrogen was 2.6 times higher in the lagoon compartment than in the stream. All other parameters were similar at both sampling sites. A rapid convergence to the chemical conditions of the local environment was observed in the transplanted bags containing water from the RS site and incubated at the LN site: conductivity inside these bags rapidly increased, from freshwater levels (0.15 mS cm^−1^) to the values registered in the lagoon *in situ* and inside the bags in the LN–LN incubations (9.3 mS cm^−1^; Figure A1 in Appendix).

#### Bacterial abundances and carbon production

**Figure 1:**
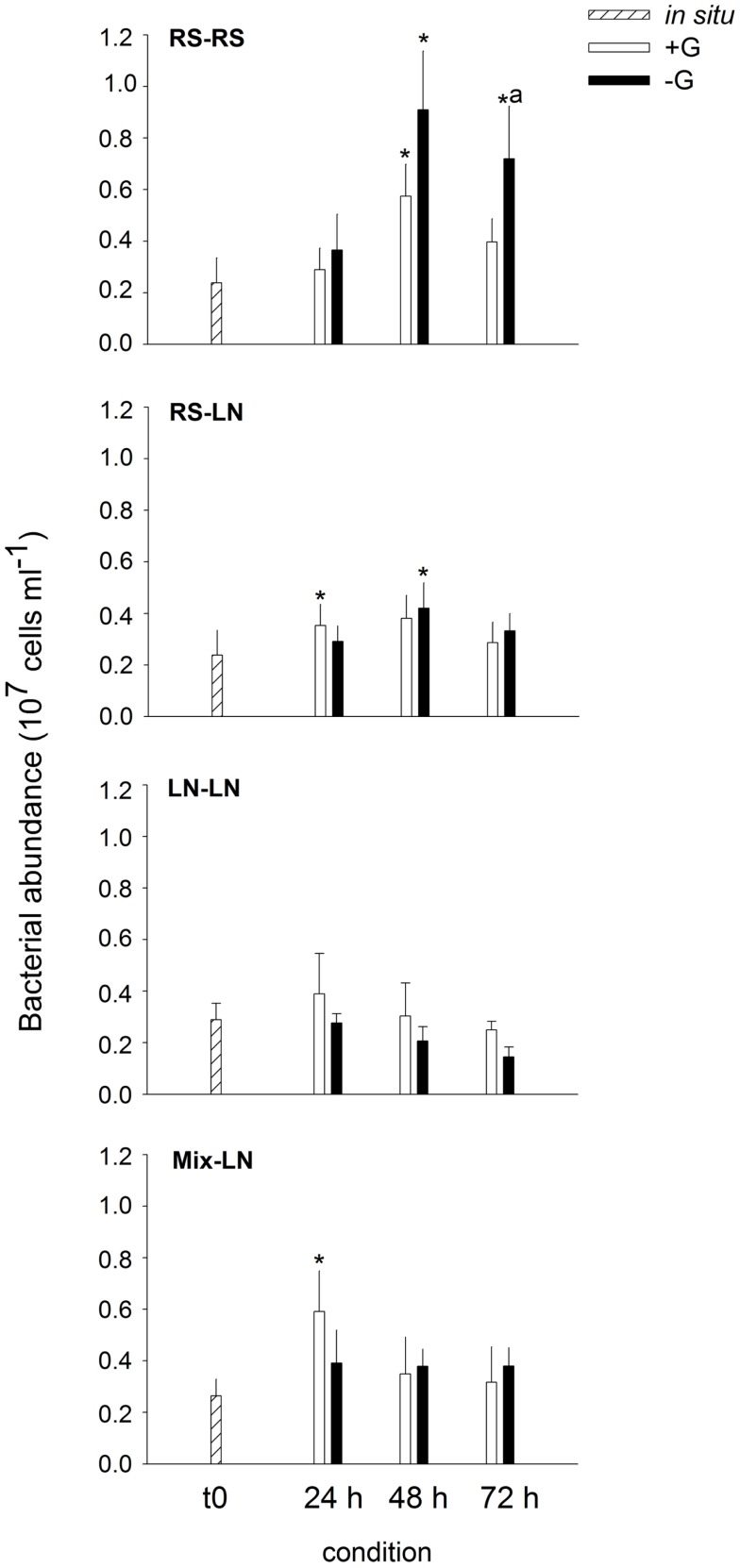
**Exp. 1, Evolution of bacterial abundance along the different incubations**. * Means a significant difference with respect to t0, ^a^ means a significant difference between the corresponding +G and −G treatments.

Bacterial *in situ* abundances were slightly higher at the LN site (Figure 1). Of the unfiltered (+G) treatments only the incubations RS–RS, RS-LN, and Mix-LN showed significant increments in bacterial abundances during the experiment (Figure 1). In the filtered (−G) treatment of RS–RS, total bacterial abundance increased by more than fourfold, and was also significantly higher than in the corresponding +G treatment (Figure 1). Bacterial abundances in the −G treatment of RS-LN was also significantly higher after 48 h of incubation than *in situ*; however, there was no significant difference between +G and −G in the RS-LN treatment.

**Figure 2:**
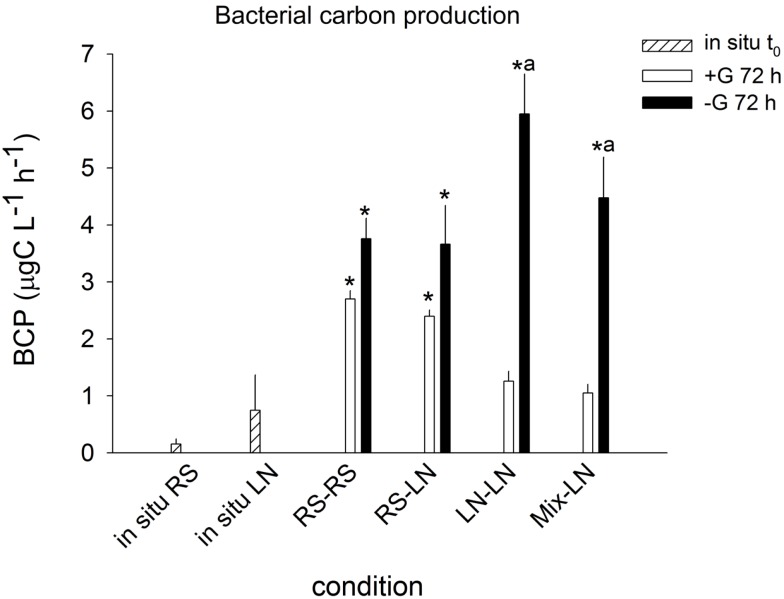
**Exp. 1, Comparison of bacterial carbon production *in situ* and after 72 h of incubation for the different treatments**. * Means a significant difference with respect to t0, ^a^ means a significant difference between the corresponding +G and −G treatments.

Lagoon samples showed higher *in situ* BCP than stream samples (0.15 ± 0.09 and 0.75 ± 0.62 μgC l^−1^ h^−1^, respectively; Figure 2), but this difference was not statistically significant. BCP in RS–RS and RS-LN rose significantly after 72 h in both, +G and −G, treatments. The carbon production values were very similar for a given treatment in both environments. For the incubations containing lagoon water (LN–LN and Mix-LN) the increment in bacterial production was only significant in the −G treatments, where the highest values were observed (Figure 2).

#### Bacterial community composition and dynamics of the main bacterial groups

**Figure 3:**
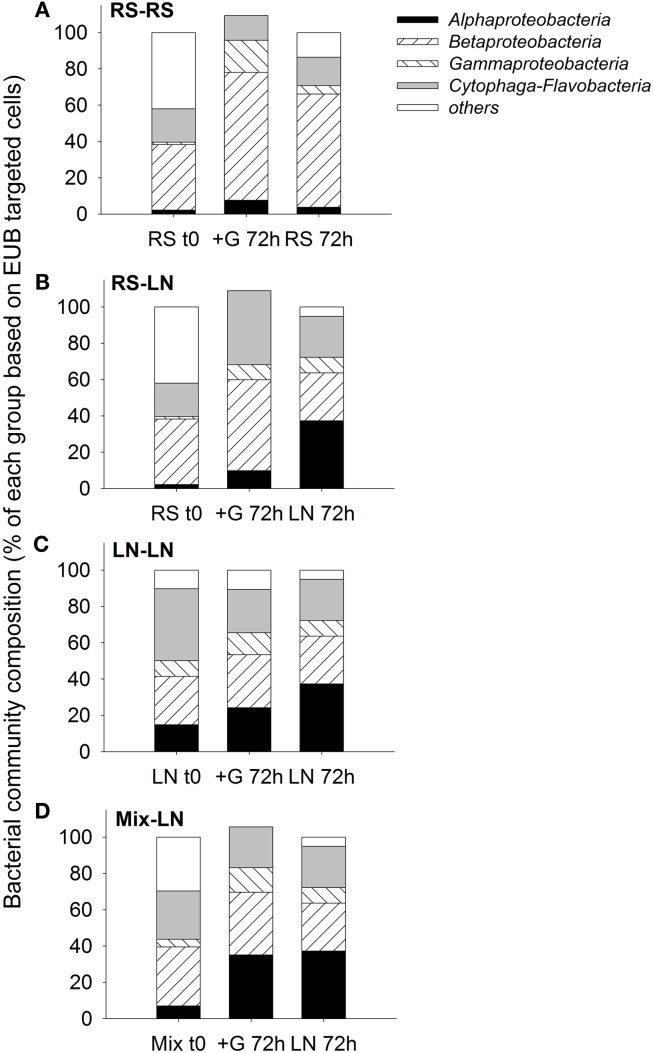
**Exp. 1, Comparison of bacterial community composition from the initial *in situ* (*t*_0_) samples with the incubated unfiltered samples (treatment +G) after 72 h, and the *in situ* community of the corresponding incubation site after 72 h of the initial *in situ* sampling. **(A)**** RS-RS incubation, **(B)** RS-LN incubation, **(C)** LN-LN incubation, and **(D)** Mix-LN incubation.

The two sampling sites differed in their *in situ* bacterial community composition. The lagoon was characterized by a higher proportion of *Alphaproteobacteria* and *Cytophaga-Flavobacteria*, while a higher proportion of *Betaproteobacteria* was found in the stream community (Figure 3). In all four treatments, after 72 h, the bacterial community composition of the unfiltered (+G) samples was more similar to the *in situ* community found at the incubation site on the corresponding sampling day than to the original community enclosed in the dialysis bags at the beginning of the incubation (Figure 3).

**Figure 4:**
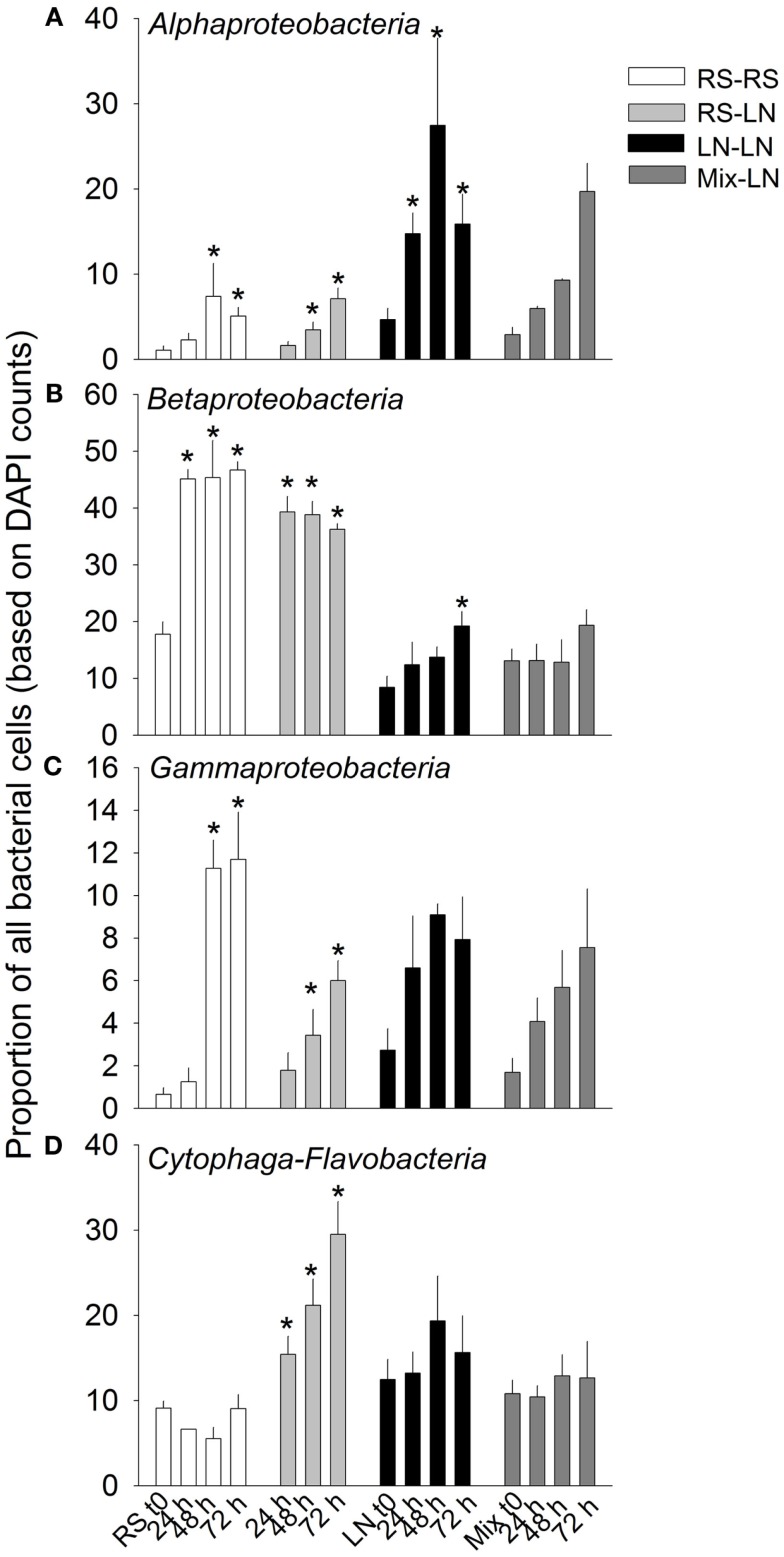
**Exp. 1, Abundance of the main bacterial groups during the incubation of the unfiltered (+G) treatments**. *Means a significant difference with respect to *t*_0_. **(A)** Alphaproteobacteria, **(B)** Betaproteobacteria, **(C)** Gammaproteobacteria, and **(D)** Cytophaga-Flavobacteria.

The main bacterial groups responded differently to the incubations (Figure 4). *Alphaproteobateria* from the RS community were similarly and moderately stimulated in RS–RS and RS-LN incubations, whereas this group was highly stimulated in the water from the LN site (LN–LN and Mix-LN) when incubated at the LN site (Figure 4A). *Betaproteobacteria* from the RS community showed a rapid and strong stimulation in both, the RS–RS and the RS-LN incubations, while their counterparts from the LN site were only belatedly and moderately stimulated in the LN–LN incubation (Figure 4B). *Gammaproteobacteria* were stimulated at all incubations. The most pronounced responses were observed when members from both, the RS site and the LN site, were incubated at their habitat of origin (RS–RS and LN–LN incubations, respectively; Figure 4C). *Cytophaga-Flavobacteria* was the group least stimulated by the treatments involving incubation in the habitat of origin. However, when members of this lineage from the RS compartment were incubated at the LN site their proportion in the community tripled with respect to the RS original community (Figure 4D).

**Figure 5:**
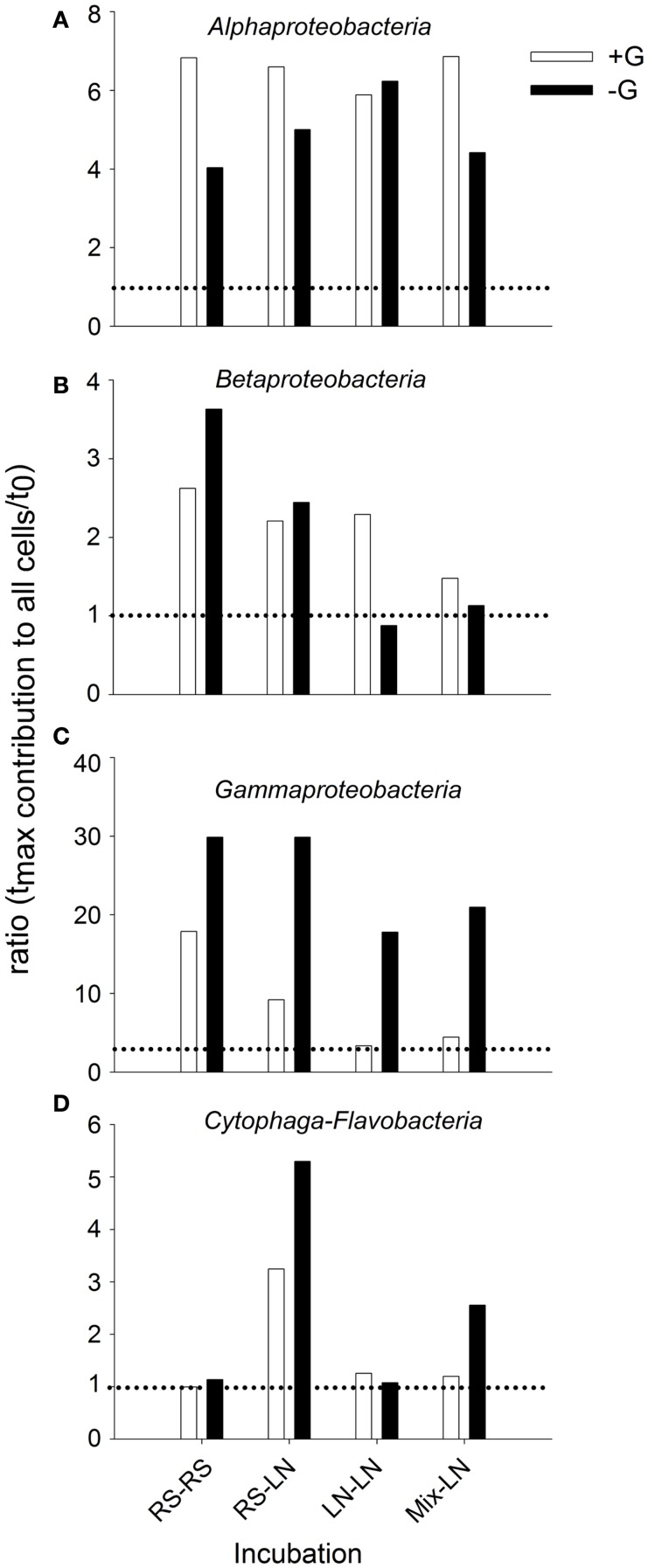
**Exp. 1, Ratios of maximal abundance over *t*_0_ abundance for each of the main bacterial groups, comparing the changes observed for the unfiltered (+G) and filtered (−G) treatments**. **(A)** Alphaproteobacteria, **(B)** Betaproteobacteria, **(C)** Gammaproteobacteria, and **(D)** Cytophaga-Flavobacteria.

When the relative abundances of the different bacterial groups were compared, they also showed different responses to the absence or presence of predators in the different incubation sites (Figure 5). With the exception of the LN–LN incubation, *Alphaproteobacteria* were generally more stimulated in the presence than in the absence of predators (Figure 5A). By contrast, *Gammaproteobacteria* were always much more stimulated in the absence of predators (Figure 5C). *Cytophaga-Flavobacteria* were also stimulated in the absence of predators, but only significantly so in the incubations where RS water was transferred to the LN site (Figure 5D). *Betaproteobacteria* of the RS community were stimulated more in the absence of predators, while they were preferentially stimulated in the presence of predators in the LN community (Figure 5B).

### Experiment 2. Gradual mixing of bacterial communities mimicking an environmental gradient

Since the sand bar was closed at the time of sampling the differences between both sampling points were relatively minor, conductivity being the most diverging variable (Table A3 in Appendix).

#### Bacterial abundance and BCP

**Figure 6:**
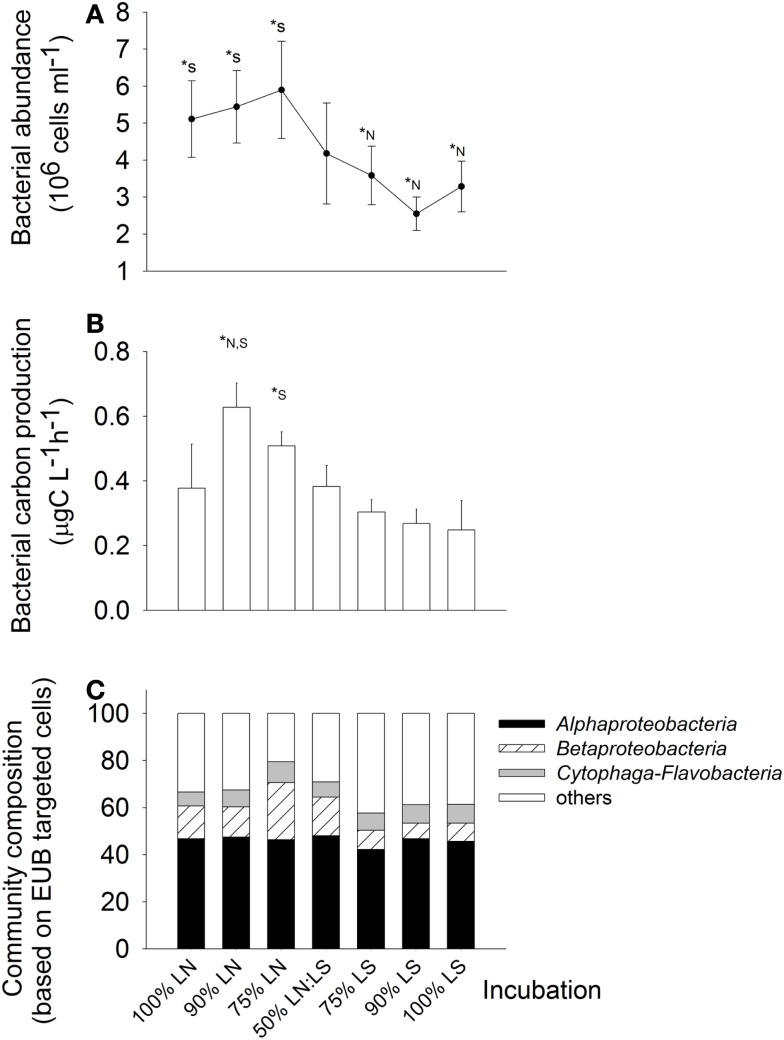
**Exp. 2, (A) Total bacterial abundance in the different treatments, (B) Bacterial carbon production for the different treatments, (C) bacterial community composition in the different treatments**. * Indicate significant differences either to the 100% LN incubation (*N) or to the 100% LS incubation (*S).

Bacteria were about twice more abundant *in situ* at the LN site (100% LN); the maximal abundances was found in the treatment of 75% LN + 25% LS, and the minimal one in the treatment of 90% LS + 10% LN (Figure 6A). *In situ* BCP was also higher at the LN site; it was stimulated by mixing of water from LN with up to 25% of LS water (Figure 6B). For example, BCP in the 90% LN treatment was significantly higher than in both, the 100% LN and LS incubations, and in the 75% LN treatment it was significantly higher than in the 100% LS incubation.

#### Bacterial community composition and tracer incorporation at single cell level

Members of the *Alphaproteobacteria*, *Betaproteobacteria*, and *Cytophaga-Flavobacteria* constituted on average 66% of all cells hybridized with the general bacterial probe, without marked differences between both sampling sites (Figure 6C). However, treatment 75% LN diverged from the others due to an increased contribution of *Betaproteobacteria* (Figure 6C).

**Figure 7:**
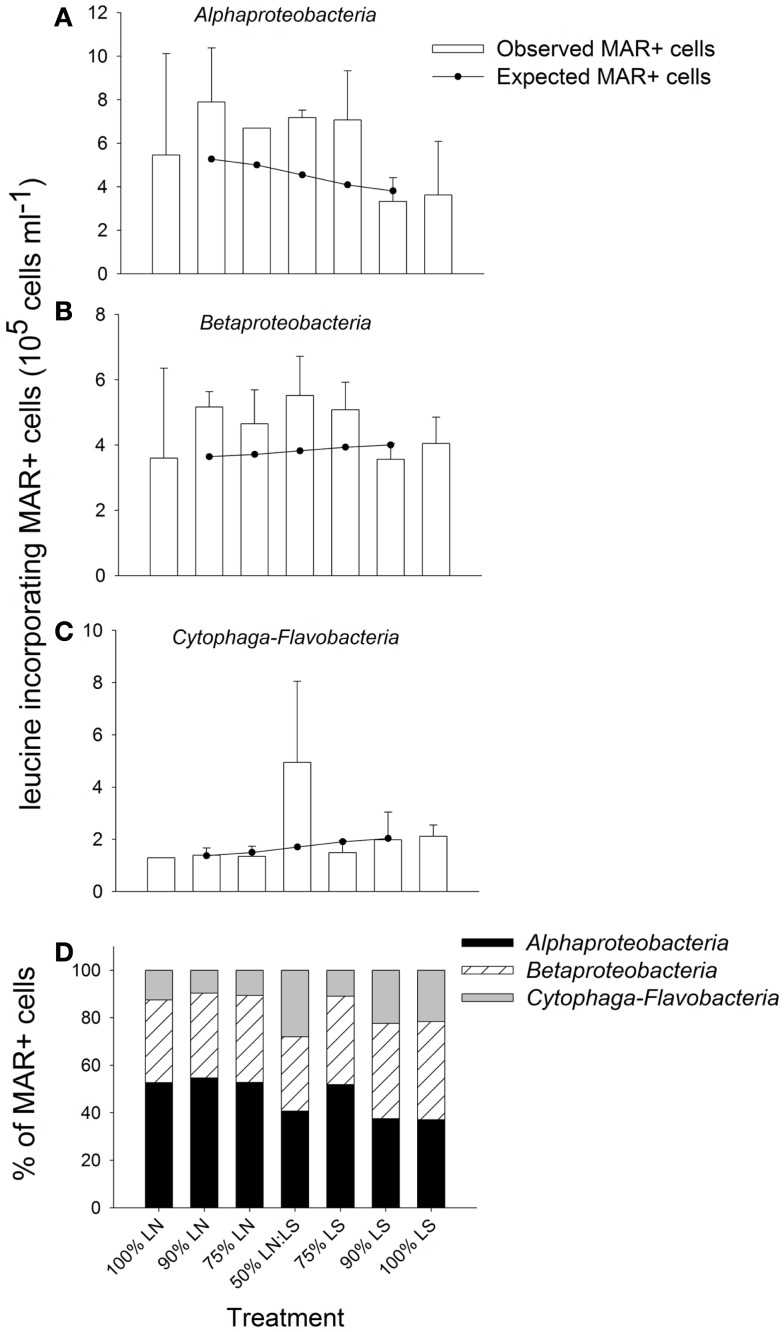
**Exp. 2, (A–C) Comparison of observed and expected numbers of MAR+ bacterial cells for each of the targeted groups and treatments**. The expected numbers were calculated based on the results for the 100% LN and LS incubations, taking into account the percentage of each water sample in the different mix treatments. **(D)** Contribution of each group to all MAR+ cells per treatment. The total number of all MAR+ cells was calculated by summing the MAR+ cells of each of the targeted groups, as they constituted the great majority of all EUB cells.

The gradual addition of LS water to LN water resulted in a stimulation of leucine incorporation by *Alpha*- and *Betaproteobacteria* (Figure 7). Both groups were particularly active in incubations containing up to 75% of LS water: in those treatments, the observed MAR+ cells were clearly above the level expected from the average activity in pure samples from both sites (100% treatments, Figures 7A,B). The lowest numbers of active cells in these bacterial groups were found in incubations of 100% LS water. *Cytophaga-Flavobacteria* showed a high stimulation by the 50:50 mixing of water from LN and LS; the number of active cells from this lineage was clearly above the number expected from mere averaging in this treatment only (Figure 7C).

*Alphaproteobacteria* were the most important contributors to all leucine-incorporating (MAR+) cells in the incubations containing up to 75% LN water, while *Betaproteobacteria* was the most important group for incubations with a contribution of LS water >50% (Figure 7D). *Cytophaga-Flavobacteria* markedly increased their contribution in the 50:50 mixture, where the respective contributions of all three groups were most similar. *Cytophaga-Flavobacteria* exhibited highest changes in contribution with incubation conditions whereas *Betaproteobacteria* was the group with the most stable contribution in all treatments.

### Experiment 3. Manipulation of carbon and nutrient sources in a mesocosms

At the sampling time the RS and LN sites exhibited conductivities of 0.5 and 8.84 mS cm^−1^, respectively. They also differed in the bacterial abundance (2.3 × 10^6^ vs. 5.2 × 10^6^, respectively).

#### Response of primary producers to the experimental manipulations

**Figure A2:**
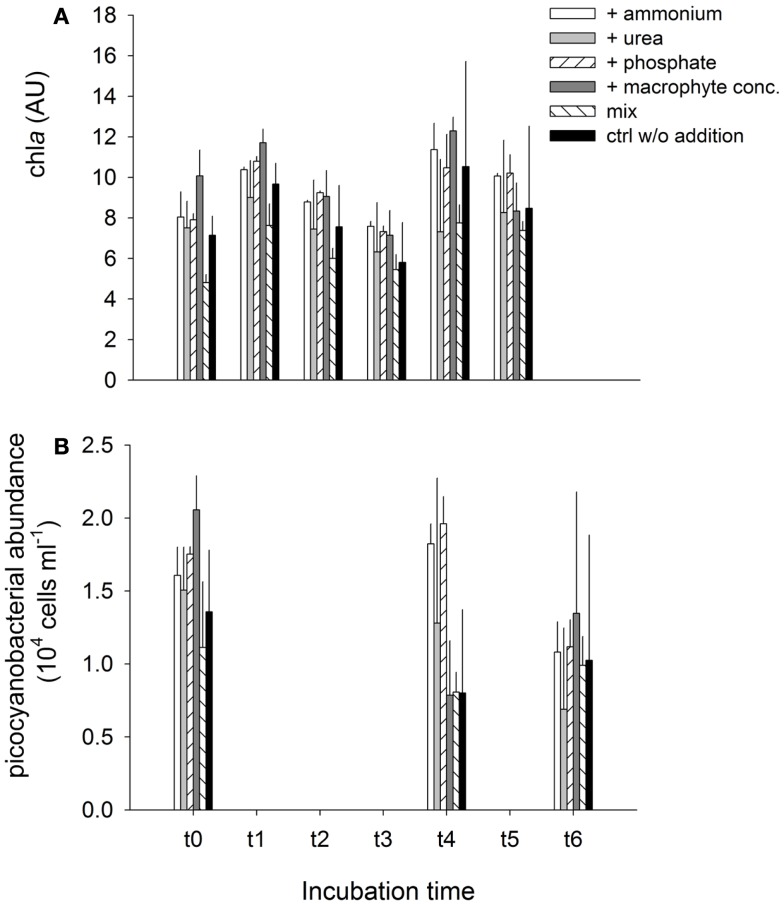
**Exp. 3, (A) Evolution of chl*a* concentration, and (B) Cyanobacterial abundance for the different treatments, during the experiment**.

Chl*a* concentration followed a similar pattern over time in all treatments, reflecting the typical circadian cycle of chl*a* synthesis (Figure A2A in Appendix). Despite some differences in chl*a* concentration between treatments (i.e., the mix of LN and RS water exhibited the lowest chl*a* values and the treatment with addition of macrophyte extract the highest), none of the experimental manipulations lead to an excessive growth (i.e., a bloom) of primary producers. The picocyanobacterial abundance dropped by an average of ca. 40% (compared to the original abundance) over time in all treatments, with the remarkable exceptions of treatment 5 (mix) in which even a moderate growth was observed (Figure A2B in Appendix).

#### Response of the bacterial community

##### Changes in abundance and community structure

**Figure 8:**
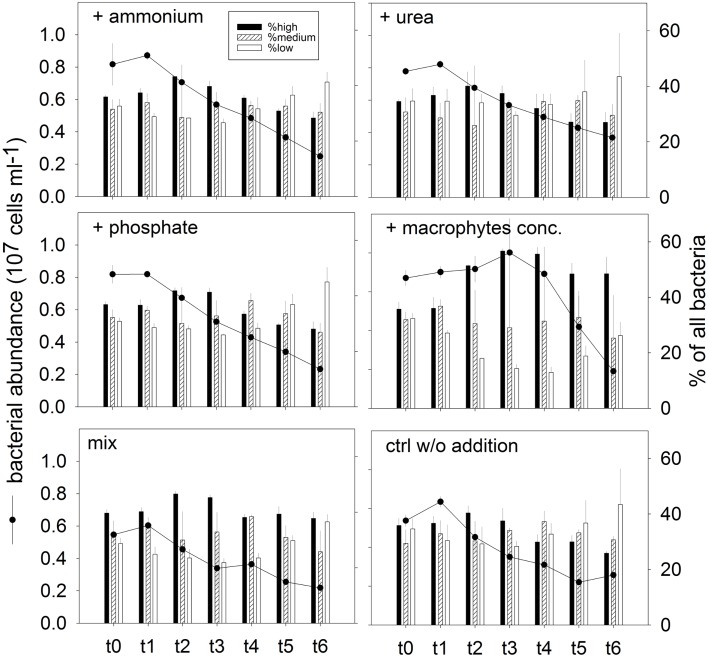
**Exp. 3, Total bacterial abundance (dots and lines) and community structure (bars), as determined by flow cytometry for the different treatments**. The different populations represented by the bars were identified in the cytograms according to their nucleic acid content.

Total bacterial number increased (average 6%) for all treatments at t1 (after 4 h; Figure 8). With the exception of treatment 4 (macrophyte conc. addition), this was the time point of highest bacterial numbers in the incubations. Maximal abundances in treatment 4 were found at t3 (16 h), being 15% higher than the respective abundance at t0 (Figure 8). A rapid decline on the bacterial abundance was observed already at t2 (8 h) in all variants except treatment 4, where the stimulation of bacterial growth was prolonged until t3 (Figure 8). At t6 (42 h, end of the experiment) the bacterial abundance was significantly reduced in all treatments, to less than half of the original value (Figure 8).

In addition, changes in phenotypic community structure were observed in terms of proportions of cells with high, medium, and low nucleic acid content (HNA, MNA, and LNA populations, respectively; Figure 8). The bacterial community at the end of the experiment was dominated by LNA cells in treatments 1 (ammonium), 2 (urea), and 3 (phosphate), and in the control treatment (Figure 8). By contrast, the dominant fraction in treatment 4 (macrophyte conc.) was constituted by HNA cells.

##### Dynamics of bacterial groups

**Figure 9:**
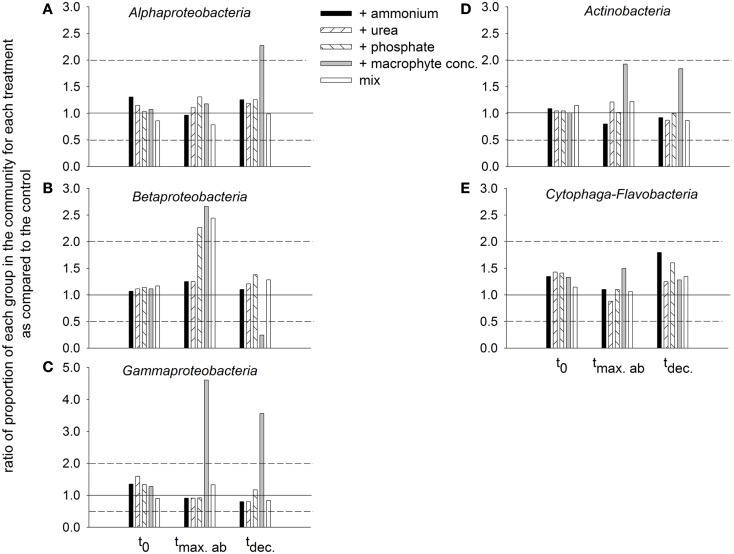
**Ratios of the proportion of the different groups in the bacterial community of each treatment in comparison to the control, at different incubation times (initial time = *t*_0_, time of maximal bacterial abundance per treatment = *t*_max.ab_, and time when bacterial abundance started to decline = *t*_dec_.)**. The solid 1:1 line marks equality to the proportion observed at the control. The dashed lines mark twofold changes with respect to the control. **(A)** Alphaproteobacteria, **(B)** Betaproteobacteria, **(C)** Gammaproteobacteria, **(D)** Actinobacteria, and **(E)** Cytophaga-Flavobacteria.

The bacterial community *in situ* and in all treatments at t0 was dominated by *Alphaproteobacteria*, followed by *Actinobacteria*, *Cytophaga-Flavobacteria*, and *Betaproteobacteria*, and finally by *Gammaproteobacteria* (data not shown). The general decrease in the dominance *Alphaproteobacteria* during incubation was substantially attenuated in the treatment with macrophyte addition (Figure 9A). *Betaproteobacteria* at the maximal abundance time increased their abundance for the treatments with phosphate, macrophyte conc., and mix. At the decline time, the strongest drop in their abundance was observed for the treatment with macrophytes, and the most attenuated drop was encountered in the treatment with phosphate (Figure 9B). *Gammaproteobacteria* at the maximal abundance time showed an explosive growth in the macrophyte addition treatment, which they still maintained at the decline time (Figure 9C). A similar pattern was observed for *Actinobacteria* (Figure 9D). *Cytophaga-Flavobacteria* exhibited a constant decline in abundance which was also attenuated in the treatment with macrophyte addition, but especially in the treatments with ammonium or phosphate addition (Figure 9E).

##### Responses of heterotrophic flagellates and viruses

**Figure 10:**
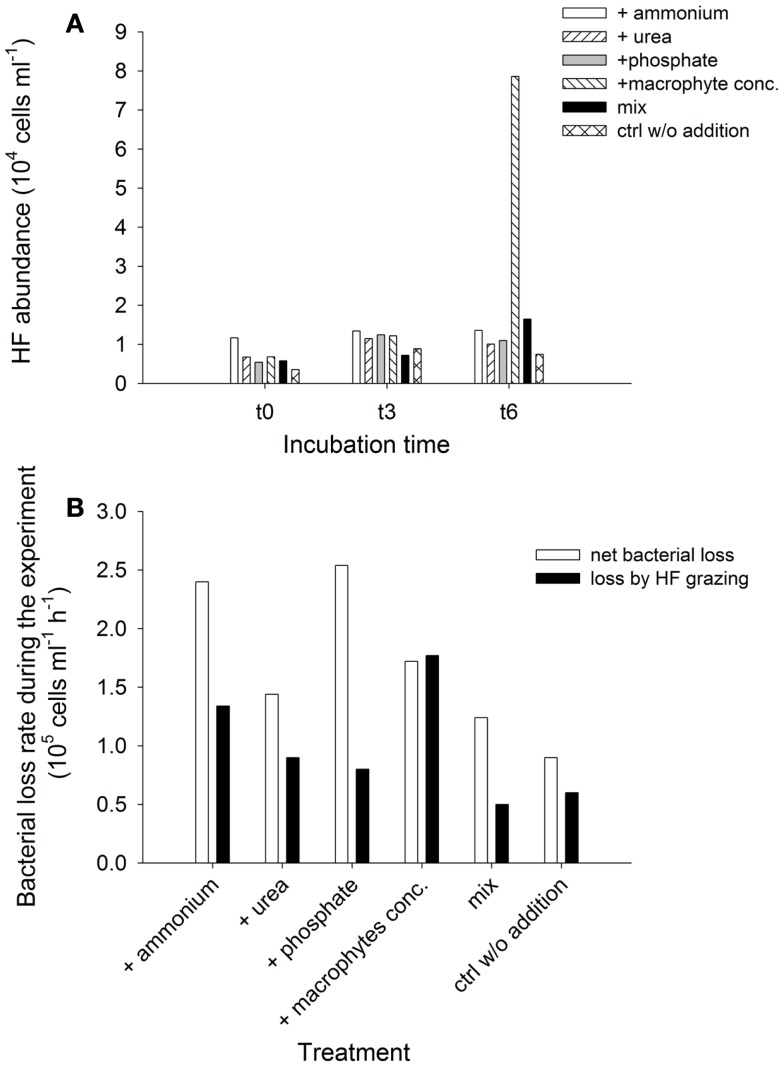
**Exp. 3, (A) Abundance of HF for selected times and treatments, (B) Comparison of net loss rate of bacterial abundance during the experiment and average loss rates due to HF grazing for each treatment**.

Heterotrophic flagellates abundances and grazing on the bacterial community were evaluated in selected samples. Although their numbers were very similar among treatments at t0 and t3, they exhibited explosive growth at the end of the experiment (t6) in the treatment with added macrophyte extract (Figure 10A). This pattern was reflected in the development of grazing rates and impact of the HF on the bacterial communities (Figure 10B). Toward the end of the experiment HF removed more than 200% of the bacterial standing stock per day in the treatment with macrophyte extract (Table 1). Bacterial abundance decreased in all treatments from t1 to t6, resulting in a negative NBP, i.e., a net loss of bacterial cell production along the experiment (Figure 10B). This loss was always higher than the HF grazing (Figure 10B), therefore HF grazing alone does not explain the drop in bacterial abundance, suggesting an alternative source of mortality for bacteria.

**Table 1:** **HF grazing rates and impact on the bacterial abundance**.

Treatment	Clearance rate (nl HF^−1^ h^−1^)			Specific grazing rate (Bact. HF^−1^ h^−1^)			Grazing impact (10^5 ^Bact/ml^−1^h^−1^)			% of daily grazing impact (% Bact/day^−1^)		
	t0	t3	t6	t0	t3	t6	t0	t3	t6	t0	t3	t6
1	2.34	1.18	1.41	22.6	6.6	3.6	2.6	0.9	0.5	47	38	46
2	1.18	2.57	1.99	9.2	15.0	4.9	0.6	1.7	0.5	19	71	48
3	1.10	2.57	1.57	8.7	13.0	3.5	0.5	1.6	0.4	14	77	41
4	1.72	0.83	1.28	15.2	9.4	4.0	1.0	1.1	3.1	28	24	241
5	1.75	1.56	1.72	9.8	6.5	3.1	0.6	0.5	0.5	24	27	68
6	0.89	2.05	2.10	6.3	10.7	6.3	0.2	1	0.5	8	44	38

Viral abundances exhibited a twofold increase in the macrophyte conc. addition treatment toward the end of the experiment (t5) compared to t0, incrementing from 1.2 × 10^8^ to 2.4 × 10^8^ particles ml^−1^. During the same period the viral abundance at the control treatment only rose from 1.3 × 10^8^ to 1.7 × 10^8^ particles ml^−1^.

## Discussion

Although the study system did not exhibit its most extreme physicochemical zonation during our experiments, we nevertheless observed distinct differences in bacterial abundance, biomass production, and community composition between sites. Moreover, despite the low phylogenetic resolution of our community analysis (phyla, classes) specific response patterns of the different bacterial taxa to environmental changes (e.g., of hydrological conditions) could be clearly distinguished.

### Hydrology affecting bacterial communities and carbon transfer to higher trophic levels

Sampling sites with contrasting salinities differed in bacterial community composition (Exp. 1, Figures 3A–C), with greater importance of *Betaproteobacteria* at lower conductivities, and of *Alphaproteobacteria*, *Cytophaga-Flavobacteria*, and *Actinobacteria* at the more brackish sites. This agrees with previous results from this system (Piccini et al., 2006) and from other estuarine environments (Kirchman et al., 2005; Alonso et al., 2010). The Northern site of the lagoon (LN) generally appears to be the zone of higher bacterial biomass production at oligohaline conditions, as compared to the meso-haline Southern sampling site (LS, Exp. 2, Figure 6A). This production is moreover seasonally dynamic, with higher values during summer (Exp. 1, Figure 2; Exp. 2, Figure 6C; Piccini et al., 2006). The difference between sites can mostly be explained by higher concentrations of nutrients and biomass of primary producers in the northern zone (Conde et al., 1999, 2000; Calliari et al., 2009), which in turn would fuel bacterial production (Cole et al., 1982).

In support of these *in situ* data, our experimental results also clearly indicated that bacterial abundances, community composition, and biomass production in Laguna de Rocha were highly dynamic, but tightly linked to hydrology: Microbial communities from the freshwater zone (RS) developed significantly different cell densities after 48 h of incubation at the brackish site (LN, Exp. 1, Figure 1), and the transplanted communities rapidly transformed to resemble the characteristic composition of the incubation zone (Exp. 1, Figure 3). Moreover, bacterial numbers, activity, and community composition changed even after only 8 h of incubation at particular degrees of mixing water from two brackish sites differing in salinity and nutrient levels (Exp. 2, Figures 6A–C; Table A3 in Appendix).

While some freshwater microbes may also thrive under brackish conditions (Piwosz et al., in press), the studied bacterial taxa nevertheless differed in their sensitivity to environmental change: At more pronounced physicochemical differences between sites (Exp. 1) all three proteobacterial groups reached higher numbers if incubated in their original habitat (either freshwater or brackish; Figures 4A–C). Moreover, their activity was enhanced at a wide range of mixing ratios of water from two brackish sites (Exp. 2, Figure 7). By contrast, the abundances and activity of *Cytophaga-Flavobacteria* were particularly stimulated by strong environmental change, i.e., when the freshwater community was transplanted to the brackish site (Exp. 1, Figure 4D) or at a 50:50 mixing ratio of water from two brackish sites (Exp. 2, Figures 7C,D). A closer look into similar studies reveals parallels to some of our findings. When Gasol et al. (2002) transplanted freshwater communities between sites, the proteobacterial groups generally reached higher abundances when incubated in their original habitat, while *Cytophaga-Flavobacteria* were stimulated by habitat change, in particular if grazing pressure was concomitantly reduced (Gasol et al., 2002). Higher abundances of *Cytophaga-Flavobacteria* have also been found in the mixing fronts of estuarine systems (Bouvier and del Giorgio, 2002; Alonso et al., 2010). We extend these findings by showing that the contribution of *Cytophaga-Flavobacteria* to BCP may also increase at such conditions (Figure 7C).

Hydrology also seemed to influence the transfer of bacterial biomass to the upper trophic levels. Bacterial abundances of the freshwater community were highly stimulated if exposed at the freshwater site in the absence of predators, whereas this was not the case if assemblages were incubated at the oligohaline site (Exp. 1, Figure 1). However, although BCP was always significantly higher in the absence of predators, the differences between treatments with and without grazers were substantially more extreme in communities from the oligohaline site (Figure 2). BCP in the brackish community incubated without grazers was in fact comparable to that in highly eutrophic systems (Furtado et al., 2001). Our results thus suggest that protistan grazing controlled bacterial abundances at the freshwater site, while other factors, e.g., viral lysis or resource limitation, prevented a translation of biomass production into higher bacterial numbers in the brackish water assemblage.

Differences in grazing pressure along (freshwater) productivity gradients have been mainly attributed to differences in grazer abundances (Gasol et al., 2002), in particularly of HF (Jezbera et al., 2005), but may also be associated with qualitative changes in the grazer community. Rapid successions of HF populations with contrasting dietary preferences have been observed in parallel with salinity changes in an estuarine system (Piwosz and Pernthaler, 2010). Hydrology also played a role in controlling both, the abundances and community structure of micro-zooplankton (>10 μm) in Laguna de Rocha (Britos, 2010). Changes in the hydrology, and therefore in salinity, might thus affect survival of protistan grazers, temporary releasing bacteria from grazing control. This in turn might eventually favor the growth of opportunistic, fast-growing bacteria (Eilers et al., 2000; Beardsley et al., 2003), as, e.g., suggested by the significant increase of *Gammaproteobacteria* in our bacterivory-free treatments (Exp. 1, Figure 5). These bacteria were most strongly affected by predation during incubations in both, freshwater and brackish environments (Figure 5C), particularly in the latter one. In fact, a release from top-down control, e.g., after marine intrusions, might be responsible for the extreme bloom of a single gammaproteobacterial species that has been observed in Laguna de Rocha (Piccini et al., 2006).

**Figure A3:**
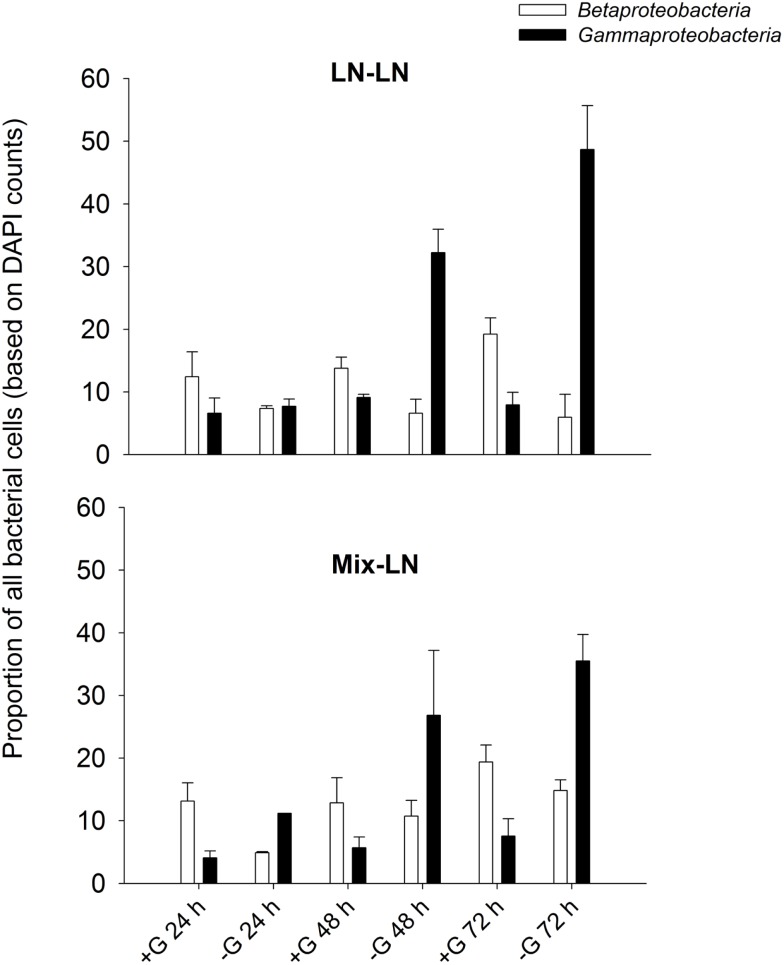
**Exp. 1, Percentages of *Beta-* and *Gammaproteobacteria* during incubations performed either with (+G) or without grazers (−G)**.

Interestingly, an apparent competition for resources between *Beta-* and *Gammaproteobacteria* in the brackish zone seemed to be modulated by grazers. At *in situ* conditions, i.e., presumably under resource shortage *Betaproteobacteria* clearly outperformed *Gammaproteobacteria* in the presence of predators, while the opposite was true in their absence (Exp. 1, Figure A3 in Appendix). By contrast, under resource-rich conditions and high predation (Exp. 3, addition of macrophyte conc., Figures 9B,C) *Gammaproteobacteria* were favored over *Betaproteobacteria*. These findings indicate that members of both groups might share a similar ecological niche, and that the outcome of competition is determined by the interplay of resource availability and intensity of grazing pressure.

*Actinobacteria* in experiment 3 experienced the lowest overall decline in all treatments, indicating that they may prevail under carbon-limited conditions and/or sustained grazing pressure. These conclusions are supported by previous observations that *Actinobacteria* are able to grow on unusual carbon sources, and might thereby even profit from the degradation activity of other bacteria (Beier and Bertilsson, 2011; Eckert et al., in press). In addition, bacteria from this lineage are typically less affected by protistan grazers, e.g., they are underrepresented in the food vacuoles of heterotrophic nanoflagellates (Jezbera et al., 2006), possibly due to their minute cell size (Posch et al., 2007) and gram-positive cell wall (Tarao et al., 2009).

In conclusion, the studied phylogenetic groups of bacteria appeared to differ in their control by bottom-up or top-down mechanisms, which in turn were tightly dependent on the hydrology of the system (summarized in Table 2).

**Table 2:** **Summary of factors controlling the abundance and activity of the main bacterial groups in the lagoon, according to whether the response was observed for limnic or brackish samples**.

	Control mechanism	α*-proteobacteria*	β*-proteobacteria*	γ*-proteobacteria*	*Cytophaga-flavobacteria*	*Actinobacteria*
Limnic	Top-down (predation)	Not affected	Strongly affected	Strongly affected	Slightly affected	Not assessed
	Bottom-up (physicochemical conditions)	Not affected	Low salinity	Low salinity	Sudden salinity change	Not assessed
			High phosphate and nitrate	High phosphate and nitrate	
Brackish	Top-down	Slightly affected	Not affected	Very strongly affected	Very strongly affected	Slightly affected
	Bottom-up	High salinity	Allochthonous DOC	Allochthonous DOC	Allochthonous DOC	Allochthonous DOC
		Allochthonous DOC	Low salinity	Low salinity	Ammonium	Urea
			Phosphate	Urea		

*The bottom-up factors shown are the ones which characterized the treatment were a positive response was observed*.

### Environmental factors linked to hydrological dynamics that possibly affect microbial assemblages

Conductivity was the most obviously changing factor between the different sampling sites; it represents a main structuring element of microbial assemblages in aquatic systems (Barberán and Casamayor, 2010), particularly of estuarine ones (Alonso et al., 2010). However the sites also differed in, e.g., nutrient concentrations (Table A3 in Appendix). The mixing of water masses during marine intrusion moreover produces strong shifts in turbidity, as well as in chlorophyll-*a* and DOC concentrations (Conde et al., 2000), thus potentially affecting bacterial responses to habitat change beyond the direct and indirect effects of salinity.

Experiment 3 helped to further disentangle the respective influences of different factors that usually change during the hydrological cycle on the bacterial communities. As no bloom of primary producers developed at any experimental condition (Figure A2 in Appendix), the responses of the bacterial community were likely direct consequences of the additions of the different nutrients and carbon sources. Although N and P have been shown to be limiting in Rocha Lagoon at specific hydrological conditions (Aubriot et al., 2004; Bonilla et al., 2005), neither nutrient significantly affected the – likely top-down induced – decline of bacterial abundances or substantially altered microbial community composition (as compared to the control treatment, Figures 8 and 9). By contrast, the macrophyte concentrate (containing both, organic carbon and nutrients) stimulated the development of a presumably highly active population of HNA cells (Figure 8) and led to conspicuous taxonomic changes in the bacterial assemblages (Figure 9).

Our results thus point to carbon availability as a major driver of changes in the abundance and composition of these microbial communities. Bacterial carbon limitation in the lagoon is also suggested by the molar ratios of dissolved organic carbon, nitrogen, and phosphorous collected during several years and phases (Conde et al., 2000, 2002; Bonilla et al., 2005). The average C:N:P molar ratio observed over a wide range of conditions was 114:15:1, suggesting a carbon proportion well below values in other comparable systems (Farjalla et al., 2006). While this proportion is arguably higher than the typical stoichiometry of bacterial biomass (50:10:1; Fagerbakke et al., 1996), a large fraction of DOC in aquatic systems is refractory to bacterial degradation (del Giorgio and Davis, 2003). Moreover, bacterial carbon demand (BCD) greatly exceeds the need for biomass production, since a substantial portion of the consumed carbon is lost via respiration (del Giorgio and Cole, 1998). The high proportions of chromophoric DOM (an indication of refractory material) of total DOM in Laguna de Rocha (Conde et al., 2000; Piccini et al., 2009), and bacterial growth efficiencies of 0.33, as estimated from the current and earlier data sets according to del Giorgio and Cole (1998) and Lopez Urrutia and Morán (2007), also indicate that bacterial growth was indeed limited by DOC. An increase of BCP accompanied by changes in BCC was also observed in Laguna de Rocha upon experimental alteration of DOC composition via photodegradation (Piccini et al., 2009), which is an important mechanism to transform refractory DOM into forms more available for microbial utilization (Lindell et al., 1995; Wetzel et al., 1995; Moran and Zepp, 1997).

It is noteworthy that the stimulation of the bacterial community by an external carbon source in exp. 3 was accompanied by a markedly higher grazing impact on the microbial community (Figure 10B), and by strong increases of the abundances of both, HF (Figure 10A) and viruses (as compared to the control). This indicates that the positive effect of additional carbon on bacterial growth was indeed rapidly transferred to other components of the microbial trophic web (Figure 10B; Table 1).

### Proposed model of functioning of the bacterial groups and hydrology, from the perspective of carbon processing

**Figure 11:**
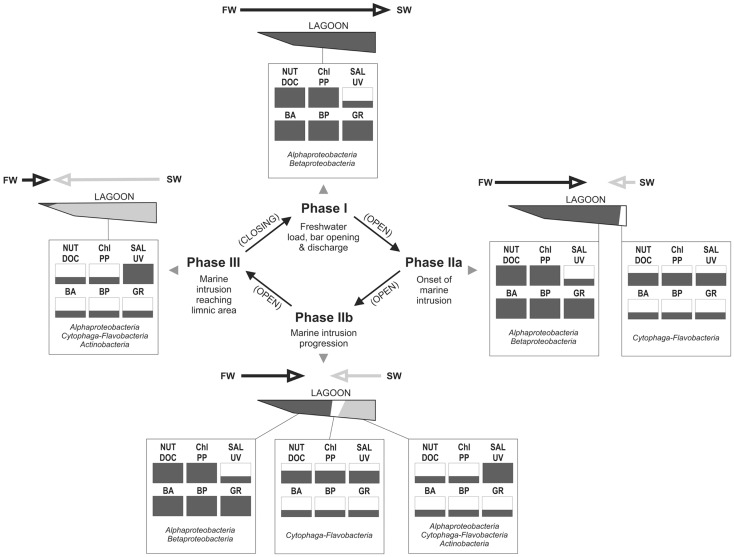
**Conceptualization of major hydrological phases occurring at Laguna de Rocha, with indication of changes in key abiotic variables and their consequences on the microbial community and processes, as derived from our data set**. Each abiotic and microbial variable is indicated as being low, medium, or high (one third, to thirds or whole box in black, respectively). Most important hydrological processes are indicated for each phase. Main arrows indicate the extent and direction of the water movement (FW, freshwater input from the watershed; SW, seawater intrusion from the coastal zone into the lagoon; Nut, nutrients (N and P); DOC, dissolved organic carbon and chromophoric dissolved organic matter; Sal, salinity; UV, UV radiation; BA, bacterial abundance; BP, bacterial production; GRAZ, protist grazing on bacteria). The names of the bacterial groups indicate which would be the main components of the bacterial community in terms of abundance and carbon production at each depicted condition.

Based on the information from this and previous studies, we propose a model for the dynamics of the bacterial groups focusing on changes in microbial carbon processing along the hydrological cycle of the lagoon (Figure 11):

In Phase I (homogeneous oligohaline brackish situation) high phytoplankton production and terrestrial input are expected across the whole lagoon as discharge proceeds from the watershed (Conde et al., 1999, 2000; Calliari et al., 2009). This situation would imply high system-wide DOC concentrations, which in turn will be similarly affected by UV radiation, as the optical characteristics of the lagoon are homogeneous (Conde et al., 2000). During this phase we predict high bacterial abundances and production, and a strong grazing pressure, as observed for the oligohaline site in our experiments (Exp. 1, Figures 1 and 2; Exp. 2, Figures 6A,C). Microbial communities are expected to be dominated by *Alphaproteobacteria* and *Betaproteobacteria* (Exps. 1–3, Figures 3, 6 and 8), which in turn would represent most of the cells contributing to BCP (Exp. 2, Figure 7).

During Phase II (marine intrusion) sudden environmental changes occur at the mixing front, derived from the mixing of water with low K, high chl*a*, high DOC, and high nutrients from the limnic zone with water with high K, low chl*a*, low DOC, and low nutrients from the marine intrusion (Conde et al., 2000). A transient stimulation of particular bacterial groups, especially of *Cytophaga-Flavobacteria*, both in abundance and contribution to BCP would be expected, as deduced from experiments 1 and 2 (Figures 4 and 7). In addition, a general relief of bacteria from grazing pressure is predicted (as observed in Exp. 1, Figure 1), with the exception of *Cytophaga-Flavobacteria* (possibly related to their higher per-cell activity; Exps. 1 and 2, Figures 5 and 7).

Once a zonation is established in Phase II, two very different habitats will form in the North and South of the lagoon (Conde et al., 2000). More terrestrial input and higher PP would be expected in the North (Calliari et al., 2009). Consequently more DOC would be available in this zone (Conde et al., 2000), albeit of a more recalcitrant quality (indicative of terrestrial origin, i.e., cDOM), which would not reach the Southern part of the lagoon (Conde et al., 2000). Due to progressive UV photodegradation (Piccini et al., 2009) this DOC is expected to increasingly stimulate carbon-limited bacterial growth (Exp. 3, Figures 8 and 9). Consequently, at this phase higher BP and bacterial abundance would be observed in the Northern oligohaline zone than in the Southern meso-polyhaline zone (as in Exp. 1 and 2, Figures 1 and 6). The community in the North would be dominated by *Alphaproteobacteria* and *Betaproteobacteria* in terms of abundance and also in terms of contribution to BCP (Exps. 1–3, Figures 3 and 6–8). In contrast, while the community would still be dominated by *Alphaproteobacteria* in the South, other important groups would be *Cytophaga-Flavobacteria* and possibly *Actinobacteria*. Although this latter group was only evaluated in the third experiment, a higher abundance of *Actinobacteria* in this zone has been previously observed after marine intrusions (Piccini et al., 2006). Moreover, there is also evidence from other aquatic systems that *Actinobacteria* adapt particularly well to strong UV radiation (Warnecke et al., 2005), a characteristic feature of the Southern zone in this phase (Conde et al., 2000). With respect to carbon transfer to upper trophic levels, a higher grazing pressure would be expected in the North (Exp. 1, Figures 1 and 2).

During phase III a homogeneous meso-polyhaline brackish lagoon is eventually established. Consequently, the whole system would develop the characteristics of the Southern zone in phase II (lower bacterial abundances and BCP, lower carbon transfer through grazing).

The best conditions for bacterial use of DOC, and carbon transfer to upper trophic levels would be expected during phase I, and during late phase II in the Northern zone. By contrast, the most pronounced carbon limitation of the bacterial community is expected to occur, during late Phase II in the South, and in the whole lagoon during the subsequent homogeneous polyhaline condition (Phase III). As the most frequent state of the lagoon is Phase II, carbon limitation would prevail in the Southern part of the lagoon during most of the hydrological cycle.

## Appendix

**Table A1:** **Oligonucleotide probes used in the different experiments**.

Probe name	Target group	Reference	Used in experiment
ALF968	*Alphaproteobacteria*	Neef (1997)	1, 2, 3
BET42a	*Betaproteobacteria*	Manz et al. (1992)	1, 2, 3
GAM42a	*Gammaproteobacteria*	Manz et al. (1992)	1, 3
CF319a	*Bacteroidetes*	Manz et al. (1996)	1, 2, 3
HGC69a	*Actinobacteria*	Roller et al. (1994)	3
EUB I-III	Bacteria	Daims et al. (1999)	1, 2

**Table A2:** **Selected samples for the different evaluations of Exp. 3**.

Treatment	Primary producers biomass and abundance of cyanobacteria	Bacterial abundance and community structure	Bacterial community composition	HNF abundance and grazing activity	Viral abundance
+Ammonium	t0, t1, t2, t3, t4, t5, t6	t0, t1, t2, t3, t4, t5, t6	t0, t1, t3	t0, t3, t6	
+Urea	t0, t1, t2, t3, t4, t5, t6	t0, t1, t2, t3, t4, t5, t6	t0, t1, t3	t0, t3, t6	
+Phosphate	t0, t1, t2, t3, t4, t5, t6	t0, t1, t2, t3, t4, t5, t6	t0, t1, t3	t0, t3, t6	
+Macrophyte concentrate	t0, t1, t2, t3, t4, t5, t6	t0, t1, t2, t3, t4, t5, t6	t0, t3, t5	t0, t3, t6	t1, t5
50:50Mix (RS:LN)	t0, t1, t2, t3, t4, t5, t6	t0, t1, t2, t3, t4, t5, t6	t0, t1, t3	t0, t3, t6	
Control without additions	t0, t1, t2, t3, t4, t5, t6	t0, t1, t2, t3, t4, t5, t6	t0, t1, t3	t0, t3, t6	t1, t5

**Table A3:** **Environmental characterization of the three sampling sites**.

Sampling point^a^	Conductivity (mS cm^−1^)	SRP (μg l^−l^)	TP (μg l^−l^)	SiO_2_ (μg l^−l^)	NH_4_ (μg l^−l^)	NO_2_ (μg l^−l^)	NO_3_ (μg l^−l^)	TN (μg l^−l^)
RS (Exp. 1)	0.2	36.6 (4.8)	36.6 (2.2)	3389.2 (367.8)	7.4 (1.6)	1.2 (1.7)	39.0 (5.1)	52.9 (24.2)
LN (Exp. 1)	9.3	25.1 (1.3)	34.3 (2.5)	2283.3 (155.6)	11.2 (10.2)	1.1 (0.2)	6.7 (0.9)	138.3 (14.5)
LN (Exp. 2)	6.5	140.0	n.d	n.d	0	7.3	117.8	n.d
LS (Exp. 2)	11.9	194.7	n.d	n.d	0	2.6	131.1	n.d

*Mean values and SD (between brackets) of the measured physicochemical characteristics at the different sampling points*.

*^a^RS, Rocha stream (freshwater); LN, lagoon north (brackish water); LS, lagoon south (brackish water); TP, total phosphorous; TN, total nitrogen; SS, suspended solids; n.d, not determined*.
